# Cake Decorating Luster Dust Associated with Toxic Metal Poisonings — Rhode Island and Missouri, 2018–2019

**DOI:** 10.15585/mmwr.mm7043a2

**Published:** 2021-10-29

**Authors:** Brendalee Viveiros, Genevieve Caron, Jonathan Barkley, Evan Philo, Sharon Odom, Jeff Wenzel, Mark Buxton, Elizabeth Semkiw, Alan Schaffer, Laura Brown, Adrienne S. Ettinger

**Affiliations:** ^1^Rhode Island Department of Health; ^2^Rhode Island Department of Health State Health Laboratory; ^3^Missouri Department of Health and Senior Services; ^4^Missouri Department of Health and Senior Services State Public Health Laboratory; ^5^Division of Environmental Health Science and Practice, National Center for Environmental Health, CDC; ^6^Rutgers Biomedical and Health Sciences, Rutgers, The State University of New Jersey, New Brunswick, New Jersey.

During 2018–2019, the Rhode Island Department of Health (RIDOH) and the Missouri Department of Health and Senior Services (DHSS) investigated cases of metal poisonings associated with commercially and home-prepared cakes decorated with products referred to as luster dust. Several types of glitters and dusts, broadly known as luster dust,* for use on prepared foods can be purchased online and in craft and bakery supply stores (*1*). Decorating foods with luster dust and similar products is a current trend, popularized on television programs, instructional videos, blogs, and in magazine articles.^†^ Some luster dusts are specifically produced with edible ingredients that can be safely consumed. Companies that make edible luster dust are required by law to include a list of ingredients on the label (*2*). Luster dusts that are safe for consumption are typically marked “edible” on the label. Some luster dusts used as cake decorations are not edible or food grade; labeled as “nontoxic” or “for decorative purposes only,” these luster dusts are intended to be removed before consumption (*3*). RIDOH (2018) and Missouri DHSS (2019), investigated heavy metal poisonings associated with commercially and home-prepared cakes decorated with luster dust after receiving reports of children (aged 1–11 years) who became ill after consuming birthday cake. Cases in Rhode Island were associated with copper ingestion, and the case in Missouri was associated with a child’s elevated blood lead level. In Rhode Island, luster dust products that had been used in cake frosting were found to contain high levels of multiple metals.^§^ These events indicate that increased vigilance by public health departments and further guidance to consumers and bakeries are needed to prevent unintentional poisonings. Labeling indicating that a product is nontoxic does not imply that the product is safe for consumption. Explicit labeling indicating that nonedible products are not safe for human consumption is needed to prevent illness from inappropriate use of inedible products on foods. Educating consumers, commercial bakers, and public health professionals about potential hazards of items used in food preparation is essential to preventing illness and unintentional poisoning from toxic metals and other nonedible ingredients.

In October 2018, the RIDOH Center for Acute Infectious Disease Epidemiology (CAIDE) investigated a report that six children aged 1–11 years became ill after attending a child’s birthday party. Symptoms of vomiting and diarrhea began 30 minutes to 10 hours after consumption of the cake, and usually lasted less than 10 hours. One person was reported to have experienced longer symptom duration and visited an emergency department for treatment. Investigators identified the birthday cake as a common food item consumed by all the children who became ill, and noted the party as the only common event. The cake, ordered from a local bakery, had been decorated with a thick layer of frosting mixed with luster dust described on the label as “gold dust” (Figure 1). CAIDE interviewed four persons who did not become ill and who reported either not eating any cake frosting (three) or eating no cake at all (one). Symptoms and illness onsets were consistent with a heavy metal poisoning (*4*), and the cake frosting was identified as the suspected food item. CAIDE obtained a picture of the cake and shared this information with the RIDOH Center for Food Protection (CFP) for further investigation.

**FIGURE 1:**
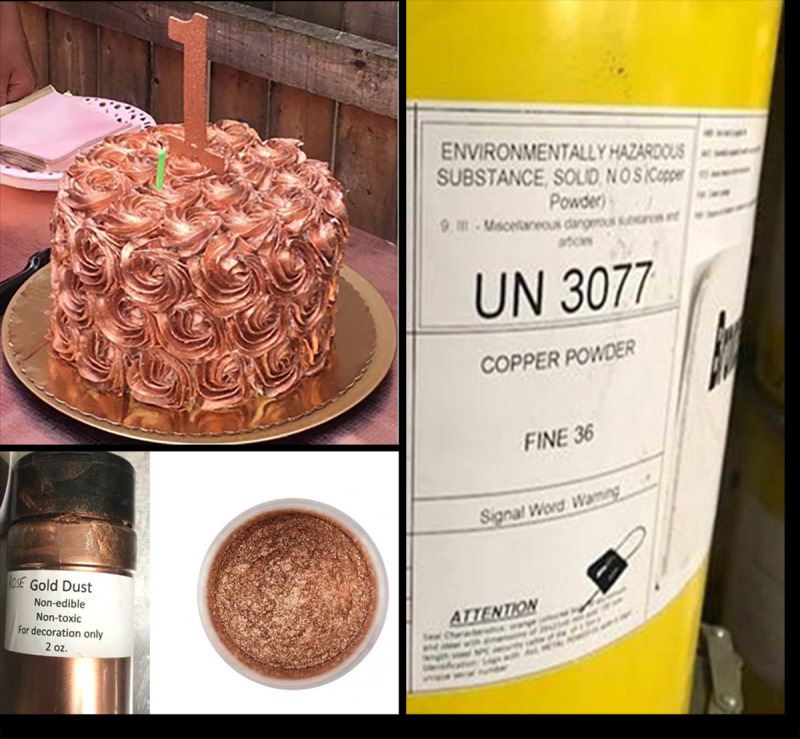
Birthday cake with rose gold dust frosting, a bottle of gold dust used for cake decorating, and industrial drums containing fine copper powder* — Rhode Island, 2018 Photos/Rhode Island Department of Health * Copper powder was commercially sold as rose gold dust.

CFP environmental health food specialists investigated the Rhode Island bakery on-site and implemented immediate control measures. In addition, a food flow analysis, conducted with CDC’s National Environmental Assessment Reporting System (NEARS) manager, traced each step in the bakery’s food preparation system to identify potential hazards and to collect evidence of contributing factors and environmental antecedents to understand and address the root causes of the foodborne illnesses. The cake ingredients and preparation process were recorded. The cake had been baked, frozen, and frosted; luster dust was added to a butter extract and painted on the cake with a brush in intervals to produce a thick layer. The luster dust applied as a decoration to the cake’s frosting was labeled as rose gold dust, and marked as “nonedible,” “nontoxic,” and “for decoration only.”

During the CFP investigation, all nonedible luster dust containers were placed under embargo. Some bottles were not clearly labeled as edible or nonedible; luster dust bottles without ingredients listed were considered nonedible. RIDOH identified and embargoed other products coated with luster dust (including chocolate pops and chocolate-covered pretzels) that were on display for retail. CFP collected several containers of luster dust, including the rose gold dust that had been used on the birthday cake, and a leftover slice of cake from the party host’s residence for chemical testing by the state health laboratory.

CFP traced three possible sources of the rose gold dust to three local companies: a cake pop bakery that sold the rose gold dust as a cake decoration, a wholesale culinary company that sold the powder to decorate cake stands, and an importer who was able to identify that the rose gold dust was fine copper powder that had been imported from a manufacturer that initially sold the powder for use as a metallic pigment for consumer goods such as floor coverings. 

Testing performed by the state health laboratory supported the suspected cause of illnesses as copper metal poisoning (*5*). Laboratory analysis identified 22.1 mg of copper per gram of rose gold frosting (nearly 900 mg of copper on the cake slice) and assigned a NEARS contamination factor of C3 (a poisonous substance accidently or inadvertently added) (*6*), which in this instance occurred as a result of misreading labels. Analysis by RIDOH of 28 other inedible luster dusts from the cake’s bakery found elevated levels of aluminum, barium, chromium, copper, iron, lead, manganese, nickel, and zinc. RIDOH visited additional bakeries and found widespread use of nonedible luster dust on food items. RIDOH issued guidance to bakeries, clarifying that labeling indicating that a product is nontoxic does not always indicate that the product is edible, and that edible luster dusts list the ingredients on the product’s label.

Subsequent to the investigation, the RIDOH Rapid Response Team presented results from the investigation at a 2018 national food safety conference. Among the attendees were the Missouri DHSS Rapid Response Team, which then disseminated information within their state to prepare response teams and alert food safety investigators about the possible risks for toxicity from luster dust products.

In May 2019, Missouri DHSS identified a cake decorating material referred to as primrose petal dust as a lead hazard during an environmental investigation of an elevated blood lead level (12 µg/dL) in a Missouri resident child aged 1 year. The child’s home, including painted surfaces and various household items, was tested for lead levels with a handheld x-ray fluorescence analyzer, which detected the presence of lead in a jar of bright yellow primrose petal dust that had been recently used in creating decorative flowers for the child’s home-baked birthday cake (Figure 2). The container for the primrose petal dust used for the cake was labeled as “nontoxic” and “made in USA” and the brand was sold by a Florida cake decorating company, which marketed it as a nontoxic color for decorating baked goods, candies, chocolate, and sugar art.

**FIGURE 2:**
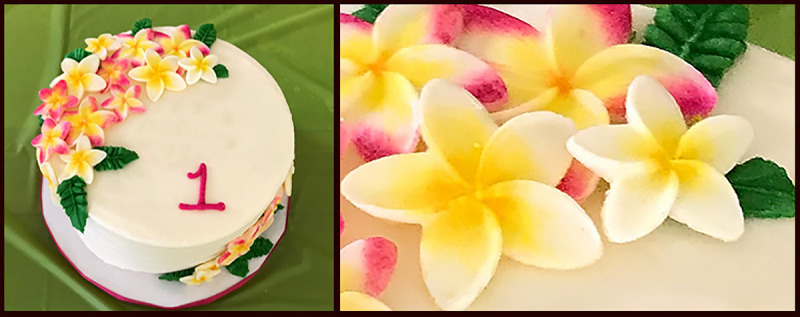
Birthday cake with icing flowers tinted with primrose petal dust used for cake decorating — Missouri, 2019 Photos/Missouri Department of Health and Senior Services

Laboratory tests conducted by the Missouri DHSS State Public Health Laboratory indicated that the primrose petal dust sample contained 250,000 ppm (25%) lead. Lead is a potent neurotoxicant, particularly in children, whose growing bodies readily absorb lead, which affects brain and other nervous system development (*7*). The Missouri DHSS issued a press release that warned consumers not to apply primrose petal dust to any food product and to immediately discard any food products that contain primrose petal dust as an ingredient. In addition, the Missouri DHSS suggested that pregnant women and parents of children who might have consumed these products consult their physician and consider having blood lead levels tested. The Food and Drug Administration (FDA) was made aware of this investigation.^¶^

## Discussion

The use of luster dust in homemade and commercially prepared goods is a popular trend; however, not all glitters are created equal. Although some glitters and dusts are edible and safe for use on food, many others are not. A recent FDA advisory (*2*) indicated that luster dust products should only be consumed if they are labeled as edible and contain a list of ingredients. By federal regulation under the Federal Food, Drug, and Cosmetic Act, the FDA requires that food additives meet certain safety and labeling guidelines (*8*). A premarket approval process is required before any listed color additive is deemed safe for its intended use or uses in or on food, drugs, or cosmetics. This premarket approval includes an assessment of toxicity based on availability of sufficient safety testing data; however, lack of such data does not deem a substance nontoxic. Even if labeled as nontoxic, these inedible products are intended for decoration only and should not be consumed. When an FDA investigation determines that a regulatory violation has occurred, the agency can take a number of enforcement actions to protect the public’s health (*8**)*. Specific enforcement activities include actions to correct and prevent violations, remove products or goods from the market, and punish offenders; this can range from issuing warning letters about violations to recommending criminal fines and prosecutions.

Labeling indicating that a product is nontoxic does not imply that the product is safe for consumption. Explicit labeling indicating that nonedible products are not safe for human consumption is needed to prevent illness and unintentional poisonings. Educating consumers, commercial bakers, and public health professionals about potential hazards of items used in food preparation is essential to preventing illness and unintentional poisoning from toxic metals and other nonedible ingredients.

**Summary**

**What is already known about this topic?**

Food decorating products known as “luster dust” are widely used on cakes and candy.

**What is added by this report?**

During 2018–2019, two states investigated heavy metal poisonings associated with commercially and home-prepared cakes using luster dusts, which were found to contain high levels of copper, lead, and other metals.

**What are the implications for public health practice?**

Labeling indicating that a product is nontoxic does not imply that the product is safe for consumption. Educating consumers, commercial bakers, and public health professionals about potential hazards of items used in food preparation is essential to preventing illness and unintentional poisoning from toxic metals and other nonedible ingredients.

## Footnotes

* Other terms include pearl dust, petal dust, disco dust, twinkle dust, sparkle dust, highlighter, and shimmer powder.

†
https://web.archive.org/web/20150905165126/http://www.craftsy.com/article/cake-decorating-history-overview-techniques

§ RIDOH measured high levels of multiple metals in luster dusts. State health departments in Rhode Island and Missouri could only confirm health issues associated with copper poisonings or elevated blood lead levels, respectively; there might have been other toxicity-associated outcomes.

¶ The Florida Department of Agriculture and Consumer Services was also informed about the investigation results.
